# Natural canal deviation and dentin thickness of mesial root canals of mandibular first molars assessed by microcomputed tomography

**DOI:** 10.1590/0103-6440202405648

**Published:** 2024-03-22

**Authors:** Carolina Alonso Amorim, Marília F. Marceliano-Alves, Isabelle Louise Gomes, José C. Provenzano, Flávio R. F. Alves

**Affiliations:** 1 Postgraduate Program in Dentistry, Estácio de Sá University, Rio de Janeiro, RJ, Brazil.; 2 Department of Endodontics, Faculty of Dentistry, Iguaçu University(UNIG), Nova Iguaçu, RJ, Brazil.; 3 Postgraduate Program in Dentistry, University of Grande Rio (UNIGRANRIO), Rio de Janeiro, RJ, Brazil.

**Keywords:** Root anatomy, danger zone, microcomputed tomography, mandibular first molar, centroid

## Abstract

The aim of this study was to assess the centralization and dentin thickness of mesial root canals of the first mandibular molars by microcomputed tomography (micro-CT). Material and methods: Ninety-nine mandibular molars of Vertucci's type IV canals were scanned by micro-CT. The mesiodistal deviation and centroid were assessed, in both mesiobuccal (MB) and mesiolingual (ML) canals, for the apical 4mm and the full canal length. Results: The dentin thickness was similar for both MB and ML canals. The narrowest thickness was in the distal wall of an MB canal (0.07mm), while the widest was found in the mesial wall of an MB canal (2.46mm). In centroid analysis, both the MB and ML canals exhibited deviations when compared to the root centroid, along the full canal length and the apical 4mm. For the MB canal, the mean deviation was 0.83mm (0.02 mm-2.30 mm) for the full canal and 0.18mm (0.01 mm-1.01 mm) for apical 4mm. Similarly, for the ML canal, the mean deviation measured 0.83 mm (0.05mm-3.99mm) for the full canal and 0.21 mm (0.01mm-1.01mm) for the apical 4 mm. Overall, deviations were observed towards the mesial of the roots, with 69% for MB and 57% for ML canals for the full canal, and 51% for MB canals within the 4 mm. The exception was the ML canal, which exhibited a higher deviation towards distal in the apical 4mm, accounting for 52% of cases. The dentin thickness was consistent between the mesial canals of mandibular molars. However, there is no centrality of mesial canals in their roots, with frequent deviation to mesial.

## Resumo

O objetivo deste trabalho foi avaliar a centralização e a espessura da dentina dos canais radiculares mesiais de primeiros molares inferiores por meio de microtomografia computadorizada (micro-CT). Material e métodos: Noventa e nove molares inferiores com canais tipo IV de Vertucci foram escaneados por micro-TC. O desvio mesiodistal e o centroide foram avaliados para os canais mesiovestibular (MB) e mesiolingual (ML), nos 4mm apicais e em todo o comprimento do canal. Resultados: A espessura da dentina foi semelhante para os canais MB e ML. A espessura mais estreita foi encontrada na parede distal de um canal MB (0,07mm), enquanto a mais larga foi encontrada na parede mesial de um canal MB (2,46mm). Na análise centroide, tanto o canal MB quanto o ML exibiram desvios quando comparados ao centroide da raiz, ao longo de todo o comprimento do canal e nos 4 mm apicais. Para o canal MB, o desvio médio foi de 0,83mm (0,02mm-2,30mm) para canal inteiro e 0,18mm (0,01mm-1,01mm) para o apical de 4mm. Da mesma forma, para o canal ML, o desvio médio mediu 0,83 mm (0,05 mm-3,99 mm) para o canal inteiro e 0,21 mm (0,01 mm-1,01 mm) para os 4 mm apicais. No geral, foram observados desvios em direção mesial das raízes, sendo 69% para canais MB e 57% para canais ML para canal inteiro, e 51% para canais MB dentro dos 4 mm. A exceção foi o canal ML, que apresentou maior desvio para distal nos 4mm apicais, representando 52% dos casos. A espessura da dentina foi consistente entre os canais mesiais dos molares inferiores. Entretanto, não há centralidade dos canais mesiais em suas raízes, com frequente desvio para mesial.

## Introduction

The root canal anatomy is one of the most complex and ancient challenges that Endodontics tries to overcome in pursuit of successful endodontic treatment ^1^ ^,^ ^2^. Its complexity may vary according to the tooth, age, gender, and ethnicity of the patient ^3^ ^,^ ^4^. Consequently, numerous treatment failures occur when these complexities lead to the involvement of regions like apical deltas, isthmus, lateral, and accessory canals. These regions present obstacles to effective cleaning, shaping, and filling of the root canal system, ultimately facilitating the persistence of pathogenic microorganisms ^5^ ^,^ ^6^.

One of the most common anatomical challenges in endodontics is the presence of curvatures in the root canal ^7^. Contrary to popular belief, there is a possibility that the root canals are not located centrally in the tooth roots ^8^ ^,^ ^9^ ^,^ ^10^ ^,^ ^11^ ^,^ ^12^ Consequently, root canals may be in closer proximity to the outer surface of the roots, such as the distal region of the mesial root, which increases the risk of deviations, ledges and perforations ^7^ ^,^ ^8^. While some prior studies have assessed dentin thickness at specific points ^9^ ^,^ ^12^, the comprehensive examination of root canal centralization within their respective roots remains relatively unexplored. This is particularly important in molars given the high frequency of operative accidents ^13^ ^,^ ^20^ ^,^ ^22^. The introduction of computerized microtomography (micro-CT) has significantly improved our understanding of these anatomical aspects ^14^ and also, allowed the centralization assessment, of the natural deviation of canals with respect to their roots. These findings may offer valuable insights for clinicians in devising instrumentation strategies to preempt intraoperative accidents.

High-resolution evaluation, such as micro-computed tomography, is the gold standard for root canal anatomy investigation because it provides non-destructive three-dimensional analyzes with a high spatial resolution that allows an accurate assessment of the morphological features ^1^ ^,^ ^6^ ^,^ ^19^ ^,^ ^20^. Then, the knowledge of those anatomical parameters, like deviation and dentin thickness of mesial root canals of mandibular first molars is important to guide clinical protocols able to promote adequate root canal preparation to contribute to the healing of periradicular tissues ^5^ ^,^ ^6^.

The objective of the present study was to assess the centralization and dentin thickness of mesial root canals of mandibular first molars by micro-CT.

## Material and methods

### Specimen selection and micro-CT scanning

The institutional ethics committee approved the study protocol. Ninety-nine extracted mandibular first molars [50 left and 49 right] with mesial root canal configuration Vertucci’s type IV ^15^, with formed apices, no previous endodontic treatment, and root resorptions were selected for the study. Buccolingual and mesiodistal radiographs, taken with a K-file #10 (Dentsply Sirona, Ballaigues, Switzerland) inserted into each root canal, confirmed the presence of two independent canals. The teeth were scanned in a micro-CT scanner (SkyScan 1174.v2; Bruker-micro-CT, Kontich, Belgium). The parameters used for the scan included 360º rotation about the vertical axis, a rotation step of 1.0, isotropic resolution of 19.9 μm, 800 mA, 50 kV, and a 0.5 mm-thick aluminum filter. The images obtained were reconstructed with the NRecon v.1.6.9 software (Bruker micro-CT, Kontich, Belgium) through axial and transversal sections of the internal structure. The quantitative evaluation by three-dimensional reconstruction of root and root canal volumes was obtained using the software plug-in three-dimensional analysis tool CTVol v. 2.2.3.0 (Bruker microCT, Kontich, Belgium).

In the CTAn v.1.14.4.1+ software (Bruker micro-CT, Kontich, Belgium) was selected the top [referring to the apical end] and the bottom [referring to the most coronary portion] of the tooth. With the top selected, through Raw Images, the line corresponding to the initial Z-position value was displayed.

After reconstruction of sections of all teeth using CTAn v.1.14.4.1+ software (Bruker micro-CT), the 99 molar images were converted to the .nrrd format in the software Image J 1.50d (National Institutes of Health, Bethesda, MD, USA) for the division of the MB and ML canals. The images were binarized, separating the images corresponding to the roots (dentin) and the MB and ML canals. The images were segmented through the linearization technique, which allows the division of the image into regions of interest (ROI), recognizing them as objects independent of each other and the background of the image. Thus, a binary image was obtained where black pixels represented the background and regions of white pixels, the object of analysis.

The ROI was duplicated using the Image J 1.50d software, and the images were subtracted. Then, the image corresponding to the root canal was obtained. All this procedure was repeated to the dentin. The saved files were converted into images sequenced in the .bmp extension for the following assessments.

### Centroid analysis

The centroid is the point associated with a geometric shape, also known as the geometric center. If the geometric shape represents a homogeneous section of a body, then the centroid coincides with the center of mass (barycentre). In cases where the body is not only homogeneous but is also subjected to a constant gravitational field, this point coincides with the center of gravity. The coordinate system for calculations of a centroid needs a differential element for integration, which can be in line, area, or volume. In the present study, the body to be studied are three-dimensional (3D) human dental roots, with a geometric region without material [root canal], fitting into the category of volume centroid ^16^ ^,^ ^23^.

From the binary images obtained by CTAn v.1.14.4.1+ software (Bruker micro-CT), the centroid was selected considering the X, Y, and Z coordinates of each sound/prepared canals or dentin model. To evaluate the deviation of the canals, only the value of centroid X, which represents deviations in the mesial [M] / distal [D] direction, was considered for the assessment.

### Quantitative analysis of data

For this analysis, the number of transversal sections of each canal obtained on micro-CT was divided by three, representing the cervical, middle, and apical thirds of the canals. The dentin thickness and canal/root ratio were measured in the middle section of each canal third. In addition, a complementary measurement was performed at 1 mm from the root apex. Both analyses were carried out using the CTAn v.1.14.4.1+ software (Bruker micro-CT) with the Measure tool option for the mesiobuccal (MB) and mesiolingual (ML) canals.

First, the root and canal's diameters were measured by drawing a straight line between the two most distant pixels on the dentin object and the measurement was recorded, after that, the same procedure was carried out for the dentin. Then, the root/canal diameters ratio of the mesiobuccal (MB) and mesiolingual (ML) canals was calculated using the CTAn v.1.14.4.1+ software (Bruker micro-CT) through the Measure tool. A straight line was drawn from one end to the other corresponding to the root/canal's diameter, and a second straight line was inserted from the distal wall to the mesial wall to pass through the center of the root/canal, which gives the root/canal diameter. This procedure was performed throughout the root length.

The results of the distances of MB and ML, concerning the centroid of the root, were positive and negative values, only to indicate which direction the deviation followed: the deviations for M were positive, and the deviations for D were negative.

**Figure 1:**
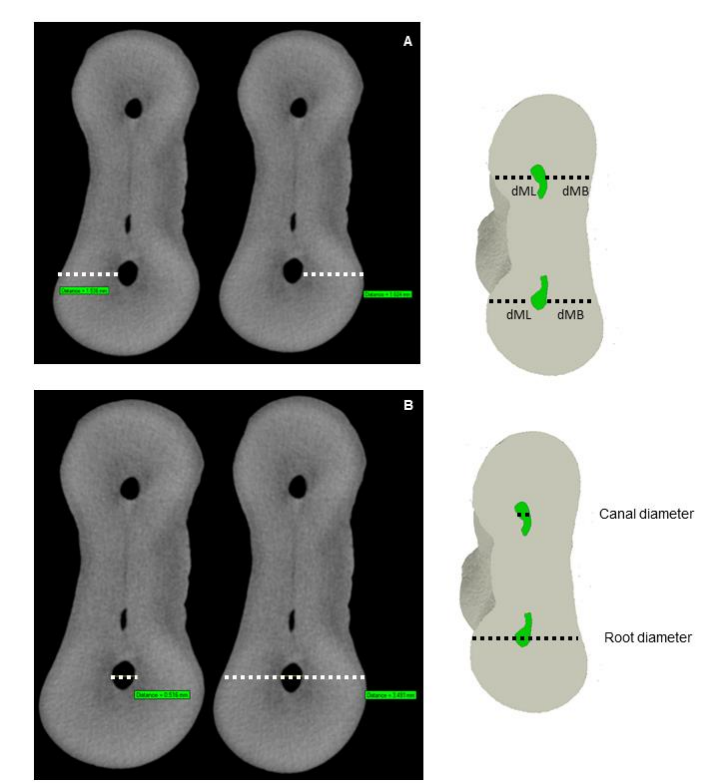
Dentin thickness measurement.

The dentin thickness was evaluated in the three-thirds of the root canal using the CTAn software (Bruker micro-CT). The Measure Tool plug-in and line option for linear measurements on the software were used, considering the distance between the two most distant pixels on the dentin object (Figure 1).

The data were analyzed and computed descriptively, calculating the mean, median, minimum, and maximum and verifying the differences between the entire length of the canal and the apical 4 mm. The frequency of deviation to M or D was also calculated. The XLSTAT-3DPlot 2018.7 program for Windows 10 (Addinsoft, New York, NY, USA) was used to construct the canal diagram from the coordinates obtained from the centroid of the mesial roots.

Finally, the CTAn software was used to create the 3D models of the canals and returned to the program CTVol v.2.2.3.0 (Bruker micro-CT) to obtain the images related to 3D models and [green], dentin (transparent gray), and centroid (black) models, allowing a more visually characterized comparison of the MD deviations between the canals and the centroids. BL deviations were not analyzed because of the absence of clinical relevance (explained in the discussion section).

## Results

The mean deviation of the center of gravity of the canals from the center of gravity of the mesial root was consistent for both ML and MB canals, measuring 0.83 mm when analyzing the entire canal and similar when only the apical region was examined (0.18 mm for the MB canals and 0.21 mm for the ML). Specifically, the mean deviation for the MB canals in the whole canal was 0.83 mm, exceeding the standard deviation of 0.71 mm, with a range from 0.02 to 2.30 mm (as shown in Table 1). In the ML root, the mean deviation was also 0.83 mm, surpassing the standard deviation of 0.76 mm, with a range from 0.05 to 3.99 mm. In the apical portion, both canals exhibited similar variations, ranging from 0.01 to 1.01 mm. The mean deviation for the MB canal in the apical region was 0.18 mm, closely matching the standard deviation of 0.15 mm. For the ML canal, the mean deviation remained at 0.21 mm, mirroring the standard deviation.

**Table 1:** Mesio-distal centre of gravity shift of the canal in relation to the root (mm^-^1)

Canal	Level	Mean (SD)	Median	Range
MB	Total canal length	0.83 (0.71)	0.64	0.02-2.30
	Apical	0.18 (0.15)	0.16	0.01-1.01
ML	Total canal length	0.83 (0.76)	0.60	0.05-3.99
	Apical	0.21 (0.21)	0.16	0.01-1.01

MB: Mesiobucal, ML: Mesiolingual, SD: Standard Deviation

**Figure 2:**
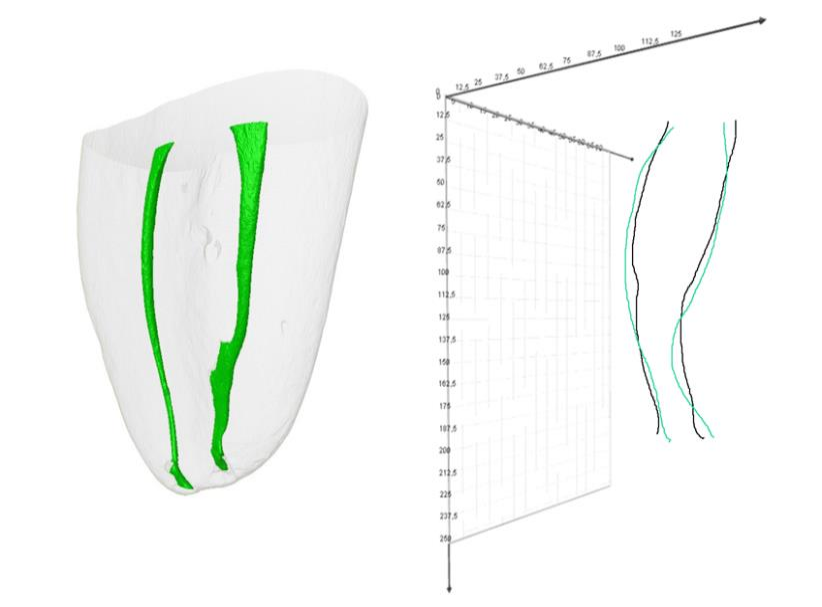
Mesial root and the center of gravity of canal (green) and root (black).

The frequency of mesial and distal deviation varied according to the canal, with mesial deviation being the most common in all analyses, except for the apical region of ML canals. When evaluating the entire canal, in both MB and ML canals, over half of the canals exhibited mesial deviations (Table 2). Notably, no data points were null, indicating that every canal demonstrated some degree of decentralization, even if minimal, regardless of the region (Figure 2).

**Table 2:** Number of canals with centre of gravity shift for mesial or distal

Canal	Level	Mesial deviation	Distal deviation
MB	Total canal length	57 (58%)	42 (42%)
	Apical	50 (51%)	49 (49%)
ML	Total canal length	68 (69%)	31 (31%)
	Apical	48 (48%)	51 (52%)

MB: Mesiobucal, ML: Mesiolingual

Analysis of dentin thickness revealed a uniform distribution between the canals (MB and ML), their walls (M and D), and across different levels (cervical, middle, apical, and 1 mm from the apex). The thinnest measurement was observed in the distal (D) wall of an MB canal (0.07 mm), while the thickest measurement was recorded in the mesial (M) wall of an MB canal (2.46 mm), as detailed in Table 3.

**Table 3:** Dentin thickness by root canal and wall (mm)

Canal	Canal wall	Level	Mean (SD)	Median	Range
MB	Mesial	Cervical	1.47 (0.35)	1.51	0.91-2.46
		Middle	1.20 (0.34)	1.09	0.61-2.14
		Apical	1.01 (0.19)	1.01	0.52-1.49
		1 mm from apex	0.77 (0.16)	0.75	0.46-1.23
	Distal	Cervical	1.25 (0.25)	1.19	0.73-1.79
		Middle	1.07 (0.23)	1.04	0.49-1.84
		Apical	0.99 (0.19)	0.95	0.61-1.51
		1 mm from apex	0.79 (0.24)	0.81	0.07-1.34
ML	Mesial	Cervical	1.42 (0.31)	1.42	0.81-2.16
		Middle	1.21 (0.28)	1.15	0.60-1.97
		Apical	1.01 (0.18)	0.99	0.61-1.79
		1 mm from apex	0.77 (0.12)	0.77	0.61-1.11
	Distal	Cervical	1.29 (0.26)	1.27	0.23-1.88
		Middle	1.15 (0.22)	1.10	0.75-1.85
		Apical	1.02 (0.20)	1.01	0.49-1.66
		1 mm from apex	0.77 (0.21)	0.80	0.21-1.25

MB: Mesiobucal, ML: Mesiolingual, SD: Standard Deviation

## Discussion

The current study marked a significant advancement by comparing the gravitational centers of root canals, in their original anatomical configuration, with those of their respective roots. To achieve this, the utilization of micro-CT was pivotal, owing to its non-destructive, high-resolution capabilities. The results have unveiled that all mesial canals do not exhibit spatial centralization concerning their roots. What's particularly striking is the degree of decentralization in millimeters, raising concerns about the potential for perforation during root canal preparation.

The MD deviation is highly relevant in clinical practice due to the small dentin thickness compared to the buccal-lingual (BL) direction, which increases the risk of root perforation or tearing during endodontic treatment^16^. On the contrary, the BL deviation is less significant in clinical terms since it encompasses a region with a substantial amount of dentin. For this reason, this deviation was not analyzed in the present study. It is important to note that MD deviations occurred in all cases, with values exceeding expectations.

Traditionally, the "danger zone" has been associated with the distal aspect of mesial roots due to its thin dentin, which predisposes the roots to strip perforations ^7^ ^,^ ^9^ ^,^ ^17^. However, recent studies demonstrated that the asymmetric position of the canals within their roots results in variable dentin thickness at different levels, often towards the mesial aspect of the root ^18^. In one study, the thinnest dentin was found in the mesial plane of the roots in 40% of the canals ^18^. The present findings challenge the conventional "danger zone" concept, indicating that mesial canals are more deviated towards the mesial aspect of the roots than the distal aspect. 

A thorough comprehension of the relationship between dentin thickness and the selection of nickel-titanium (NiTi) instruments is crucial for the success of root canal treatments. The choice of NiTi instruments should align with the dimensions of the root canal, taking perforation risk into account. In this study, the average dentin thickness in the apical third of the root canal was 1 mm, and it decreased to 0.77 mm near the apex on average. While this thickness might be considered safe, it is contingent upon the instrument selected for root canal preparation. Another study evaluated the apical dimensions of mandibular molar mesial root canals using micro-CT and compared them with the dimensions of available NiTi instruments. Based on the mean anatomical diameters, the suitable instrument dimensions would be 40/.10 for MB canals and 45/.08 for ML canals to adequately address the maximum canal surface area. However, this approach might be risky given the dentin thickness. Ideally, root canal preparation should be customized for each canal. A study employing cone-beam computed tomography (CBCT) assessed unprepared surface areas in the apical 4-mm segment of mandibular premolar root canals in human cadavers ^21^. Customized preparation encompassed a substantial portion of the root canal's apical 4 mm (mean > 90%) and necessitated final instruments one size larger than the initial largest canal, following a conservative approach. Consequently, clinicians should diligently select NiTi instruments that align with the dimensions of the root canal to mitigate the risks of overpreparation and perforation. Root fractures are unpredictable events that can transpire anywhere within the root structure. A previous study ^22^ confirmed that multiple factors exert influence on the susceptibility and fracture patterns, with one variable often prevailing over others. Nonetheless, a substantial portion of fracture susceptibility stems from intrinsic root and canal morphology, including dentin thickness, canal shape, size, and external root configuration, which are beyond the clinician's control ^(^ ^21^. In context, endodontic preparation should be as conservative as possible, consistent with proper cleaning and shaping, especially considering the natural canal deviation with the root, as disclosed in the present study. Clinicians should carefully evaluate the dentin thickness and make prudent choices regarding NiTi instruments to minimize the risks of overpreparation and perforation.

One limitation of the present study is that the internal root anatomy may be modified by local factors such as patient age, gender, occlusal dysfunctions, and periodontal disease and these factors could be considered in further research ^(^ ^15^.

## Conclusions

In mandibular first molars, the mesial canals exhibit a lack of centralization with the external anatomy of their respective roots. When considering the entire length of the canals, the mean deviation is substantial (0.83 mm for both mesiobuccal (MB) and mesiolingual (ML) canals). Throughout the full canal extension, the most frequent deviation occurred towards the mesial aspect. However, in the apical region, the deviation frequency was evenly distributed between the mesial and distal directions. The average dentin thickness in the apical third of the root canal measured 1 mm, reducing to 0.77 mm near the apex. These thickness values displayed a consistent similarity between the MB and ML canals, as well as their mesial (M) and distal (D) walls.
